# A case report of cerebellar hemangioblastoma simulated brain metastasis shown by magnetic resonance imaging

**DOI:** 10.1097/MD.0000000000037162

**Published:** 2024-02-09

**Authors:** Jiaxing Xue, Chenlong Mo

**Affiliations:** a Department of Neurosurgery, People’s Hospital of Dingzhou, Dingzhou, China.

**Keywords:** brain metastasis, case report, hemangioblastoma, magnetic resonance imaging

## Abstract

**Rationale:**

Hemangioblastomas occur both sporadically and as an important component of von Hippel-Lindau (VHL) disease. The typical MRI features of hemangioblastoma are cysts with enhanced cystic wall nodules in the cerebellum or lesions with uniform enhancement on the surface or inside the spinal cord. If there is edema around hemangioblastoma, it is easy to be misdiagnosed as brain metastasis on MRI.

**Patient concerns:**

A 41-year-old male patient was found to have a lump in the pancreas during a health examination 3 months ago. Subsequently, the patient underwent surgical treatment. The postoperative pathology suggests that the pancreatic lesion is a neuroendocrine tumor. The patient subsequently came to our hospital for consultation on further treatment plans. Abnormal signals were found in the right cerebellum during pituitary magnetic resonance imaging (MRI) before the development of a treatment plan for neuroendocrine tumors. Subsequently, the patient underwent cerebellar mass resection surgery. The pathological result after the surgery was hemangioblastoma.

**Diagnosis:**

The patient underwent surgery to remove the tumor and was diagnosed with hemangioblastoma by pathological examination. Subsequently, the patient’s genetic testing results confirmed the diagnosis of VHL syndrome.

**Interventions:**

The patient underwent cerebellar mass resection surgery.

**Outcomes:**

The patient recovered after surgical resection.

**Lessons:**

In this report, we emphasize the atypical MRI manifestations of hemangioblastoma. For patients with VHL syndrome-related hemangioblastoma, genetic testing is necessary for the patient and their family members.

## 1. Introduction

Hemangioblastomas are uncommon, slow-growing tumors of the central nervous system, which most commonly occur in the cerebellum, brainstem, or spinal cord.^[1–3]^ They account for approximately 4 percent of all spinal cord tumors and 7 to 10 percent of tumors arising in the posterior fossa in adults.^[4]^ Hemangioblastomas occur both sporadically and as an important component of von Hippel-Lindau (VHL) disease.^[3,4]^ VHL disease is an inherited, autosomal-dominant syndrome manifested by a variety of benign and malignant tumors, including hemangioblastomas, retinal angiomas, endolymphatic sac tumors, renal cell carcinoma, pheochromocytomas, pancreatic cysts, and neuroendocrine tumors. Hemangioblastomas may occur either sporadically or as a manifestation of von Hippel-Lindau (VHL) disease.^[5]^ Although approximately 75% of all hemangioblastomas appear to be sporadic, some of these may represent occult cases of VHL that can be detected if patients are appropriately screened for germline VHL pathogenic variants.^[6]^ Hemangioblastomas can cause local symptoms by compression of neural structures, bleeding, or paraneoplastic complications.^[7]^ In patients with VHL disease, asymptomatic hemangioblastomas are diagnosed based on imaging surveillance.

The majority of hemangioblastomas occur in the posterior fossa, with 80 percent in the cerebellar hemispheres and 15 percent in the cerebellar vermis.^[8]^ The characteristic appearance on magnetic resonance imaging (MRI) is either that of an intra-axial cystic mass with an enhancing mural nodule abutting the pia or a solid, intensely enhancing mass, often with flow-voids from dilated vessels next to or within the tumor.^[9]^ Smaller hemangioblastomas (<10 mm) may be isointense on T1-weighted images and hyperintense on T2-weighted images, with homogeneous contrast enhancement. The preferred diagnostic procedure is gadolinium-enhanced MRI.^[10]^ A computed tomography (CT) scan of the neuraxis is not an adequate diagnostic procedure because bone artifacts may obscure small tumors in the posterior fossa or spinal canal. For patients who cannot undergo an MRI, conventional angiography together with CT scanning are alternative diagnostic tests to define the location and vasculature feeding the hemangioblastoma.^[3]^

The prognosis of patients with total tumor resection is good, with a recurrence rate of 12% to 14%. Recurrent patients can also achieve good outcomes through further surgery.^[11]^ The surgical risk of “blood mother” is relatively high, especially if there is no correct understanding and evaluation of the tumor before surgery, once major bleeding occurs during surgery, the prognosis may be poor.^[11,12]^ The surgical efficacy of sporadic and cystic hemangioblastoma is relatively satisfactory, while the treatment of solid, familial, and multiple HB is still difficult, especially when located in the brainstem and spinal cord.^[13]^

If there is edema around hemangioblastoma, it is easy to be misdiagnosed as brain metastasis on MRI. Recently, a case of cerebellar hemangioblastoma simulated brain metastasis shown by magnetic resonance imaging was admitted to our hospital, which is reported as follows.

## 2. Case report

**Figure 1.**
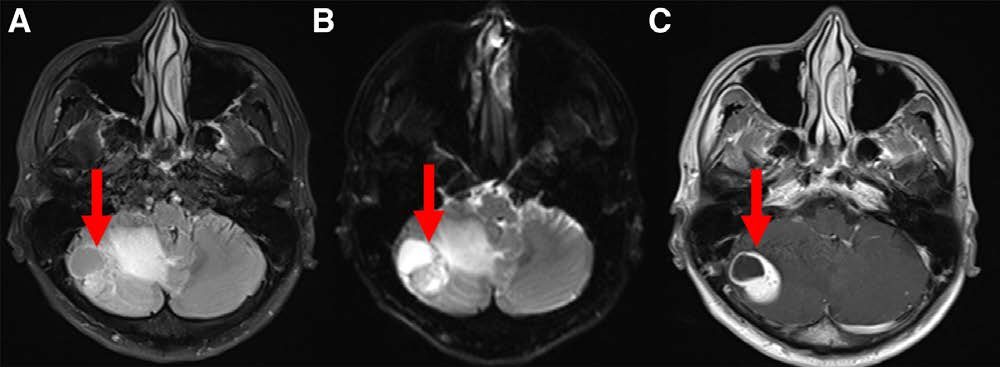
Brain MRI image of the patient. The T2WI/FLAIR (A), of the right cerebellar hemisphere mass (size, 3.2 × 2.4cm) is mixed with high signal intensity, accompanied by large edema signals at the edge, DWI (B) shows equal/low signal intensity, and the enhanced scan shows significant enhancement (C). MRI = magnetic resonance imaging.

A 41-year-old male patient was found to have a lump in the pancreas during a health examination 3 months ago. Subsequently, the patient underwent robot-assisted laparoscopic exploration under general anesthesia and underwent radical pancreatectomy and splenectomy. The postoperative pathology suggests that the pancreatic lesion is a neuroendocrine tumor, with G3 and Ki67 being 23%, SSTR2(-), PD-1(-), PD-L1(22C3)(CPS = 0), PD-L1(22C3Neg)(-), and the tumor size being 10 × 7 × 5cm. The patient subsequently came to our hospital for consultation on further treatment plans. Abnormal signals were found in the right cerebellum during pituitary magnetic resonance imaging (MRI) before the development of a treatment plan for neuroendocrine tumors (Fig. 1). The serum CA724 slightly increased to 12.7 U/mL (reference range, 0–6.9 U/mL). Serum NSE, progastrin-releasing peptide (ProGRP), CEA, CA199, and CA125 are normal.

The T2WI/FLAIR (Fig. 1A), of the right cerebellar hemisphere mass (size, 3.2 × 2.4cm) is mixed with high signal intensity, accompanied by large edema signals at the edge, DWI (Fig. 1B) shows equal/low signal intensity, and the enhanced scan shows significant enhancement (Fig. 1C). Based on the current case information and MRI results, we speculate that the abnormal signal in the right cerebellum may be a neuroendocrine tumor with brain metastasis. Subsequently, the patient underwent cerebellar mass resection surgery. The pathological result after the surgery was hemangioblastoma. After multidisciplinary consultation and discussion, the possibility of VHL syndrome was considered.

Through additional inquiries about the patient’s medical history, we found that the patient had a history of surgery for clear cell renal cell carcinoma of the right kidney 15 years ago. Subsequently, the patient provided an important family history, indicating that his mother was also a patient with VHL syndrome and had undergone surgery for cerebral hemangioblastoma many years ago. Based on the current situation, we suggest that relatives of the patient undergo VHL gene testing. One month later, the results showed that the patient’s daughter also suffers from VHL syndrome. Subsequently, the patient’s genetic testing results confirmed the diagnosis of VHL syndrome.

## 3. Discussion

Hemangioblastoma, also known as vascular reticuloma in some cases, is an uncommon central nervous system tumor that grows slowly and is more common in the cerebellum, brainstem, or spinal cord.^[8]^ This disease accounts for approximately 4% of all spinal cord tumors and 7% to 10% of adult posterior fossa tumors.^[14]^ Hemangioblastoma can be both sporadic and an important component of VHL syndrome. VHL syndrome is an autosomal dominant inherited syndrome characterized by various benign and malignant tumors, including hemangioblastoma, retinal hemangioma, endolymphatic sac tumor, renal cell carcinoma, pheochromocytoma, pancreatic cyst, and neuroendocrine tumor.^[4]^

Hemangioblastoma can cause neurological dysfunction due to direct compression or tumor-related bleeding.^[15]^ The specific dysfunction caused by direct compression depends on the tumor site, which may include dizziness/dizziness, cerebellar ataxia, oculomotor nerve dysfunction, dysphagia, motor weakness, or sensory defects. Patients with spinal cord hemangioblastoma often seek medical attention due to pain.^[15,16]^

The preferred diagnostic method is gadolinium-enhanced MRI. CT scanning of the central nervous system is not an appropriate diagnostic method, as bone artifacts may mask smaller tumors in the posterior cranial fossa or spinal canal. For patients who cannot undergo MRI, another diagnostic test is routine angiography combined with CT scanning to determine the location and vascular supply of hemangioblastoma. The typical MRI features of hemangioblastoma are cysts with enhanced cystic wall nodules in the cerebellum or lesions with uniform enhancement on the surface or inside the spinal cord.^[3]^ These characteristic manifestations do not have diagnostic significance and still need to be differentiated from astrocytoma, ganglioneuroglioma, or metastatic tumor.

The main reason why this case of hemangioblastoma was misdiagnosed as a metastatic tumor was due to the patient’s history of malignant tumors and the extensive edema at the edge of the lesion. This evidence all points to the diagnosis of metastatic tumors. Hemangioblastoma generally does not show large areas of edema around it.

For patients without known VHL disease who present with a single suspected hemangioblastoma, we suggest surgery rather than observation both for treatment and to establish the diagnosis (Grade 2C).^[17]^ Radiation therapy is an option for recurrent or residual disease if the risk of reoperation is high. For patients with known VHL disease, treatment decisions should be individualized and involve multidisciplinary input from neurosurgery, oncology, and radiation oncology. Observation is appropriate in many patients with asymptomatic tumors, and the hypoxia-inducible factor-2alpha inhibitor, belzutifan, is now an option that may delay or avoid the need for surgery or radiation therapy.^[18,19]^

## 4. Conclusion

In conclusion, we report a rare case of hemangioblastoma misdiagnosed as a metastatic tumor. This case suggests that the MRI of hemangioblastoma may also present as extensive edema at the edges. For patients with VHL syndrome-related hemangioblastoma, genetic testing is necessary for the patient and their family members.

## Acknowledgments

We thank the patient and their family for their participation in this study. There is no conflict of interest for all authors.

## Author contributions

**Data curation:** Jiaxing Xue.

**Methodology:** Jiaxing Xue.

**Supervision:** Chenlong Mo.

**Writing – original draft:** Jiaxing Xue.

**Writing – review & editing:** Chenlong Mo.
